# Agreement in Measures of Macular Perfusion between Optical Coherence Tomography Angiography Machines

**DOI:** 10.1038/s41598-020-65243-2

**Published:** 2020-05-20

**Authors:** Wei Dai, Miao-Li Chee, Shivani Majithia, Cong Ling Teo, Sahil Thakur, Ning Cheung, Tyler Hyungtaek Rim, Gavin S. Tan, Charumathi Sabanayagam, Ching-Yu Cheng, Yih-Chung Tham

**Affiliations:** 1 Singapore Eye Research Institute, Singapore National Eye Centre, Singapore, Singapore; 2 Ophthalmology & Visual Sciences Academic Clinical Program (Eye ACP), Duke-NUS Medical School, Singapore, Singapore; 3 Department of Ophthalmology, Yong Loo Lin School of Medicine, National University of Singapore, Singapore, Singapore

**Keywords:** Imaging, Medical imaging

## Abstract

We evaluated the agreements in foveal avascular zone (FAZ) area and vessel density (VD) parameters (within the superficial capillary plexus region), between two widely used optical coherence tomography angiography machines. Participants who attended the Singapore Malay Eye Study III between 29th March and 6th August 2018, were enrolled in this study. Participants underwent fovea-centered 6×6-mm macular cube scan, using both AngioVue and Cirrus HDOCT machines. Scans were analyzed automatically using built-in review software of each machine. 177 eyes (95 participants) without retinal diseases were included for final analysis. Mean FAZ area was 0.38 ± 0.11 mm^2^ and 0.30 ± 0.10 mm^2^, based on AngioVue and Cirrus HDOCT, respectively. Mean parafoveal VD was 0.50 ± 0.04 in Angiovue, and 0.43 ± 0.04 in Cirrus HDOCT. Cirrus HDOCT measurements were consistently lower than those by AngioVue, with a mean difference of −0.08 (95% limits of agreement [LOA], −0.30–0.13) mm^2^ for FAZ area, and −0.07 (95% LOA, −0.17–0.03) for parafoveal VD. Intraclass correlation coefficients for FAZ area and parafoveal VD were 0.33 and 0.07, respectively. Our data suggest that agreements between AngioVue and Cirrus HDOCT machines were poor to fair, thus alternating use between these two machines may not be recommended especially for follow up evaluations.

## Introduction

Optical coherence tomography angiography (OCTA) is a novel non-invasive technology used to image the retinal microvasculature. Since its approval by the Food & Drug Administration in 2016, OCTA has gained rapid usage in clinical practice^1^.

Compared to conventional fluorescein angiography and indocyanine green angiography, OCTA has several advantages. First, OCTA can visualize retinal blood vessels with depth resolution in a faster and non-invasive manner^2^. In addition, OCTA can image retinal blood vessels at different layers, providing new opportunities for clinicians to detect subclinical changes in eye diseases such as diabetic retinopathy (DR)^3^, glaucoma^4^, and AMD^5^.

The Cirrus High Definition OCT (HDOCT)^TM^ (Carl Zeiss Meditec) and Optovue (AngioVue^TM^) are the two commonly used OCTA modalities^1^. However, the agreement in measurements between these two instruments had yet been evaluated comprehensively. Given that imaging algorithms and automatic segmentation methods vary among different manufacturers, it is important to ascertain this aspect so as to further aid the interpretation of OCTA parameters obtained from different machines.

Hence, in this study, among eyes without retinal diseases, we evaluated the agreement in measurements between two widely used OCTA machines. Findings from this study will provide useful information on the appropriateness of alternating the usage of different OCTA machines in clinical practice and multi-site research trials.

## Methods

### Study population

We conducted a cross-sectional study including 147 participants who attended the Singapore Malay Eye Study (SiMES) III between 29th March and 6th August 2018. The detailed methodology of SiMES had been reported elsewhere previously^6,7^. This study was approved by the SingHealth Centralized Institute Review Board. Written informed consent was obtained from all participants before enrolment and the conduct of the study adhered to the Declaration of Helsinki.

### OCTA image acquisition

After pupil dilation, OCTA image acquisition of the macula was performed using AngioVue (RTVue-XR SD-OCT, Optovue, Inc., Freemont, CA, USA) and Cirrus HDOCT (Model 5000, Carl Zeiss Meditec, Dublin, CA. USA). The imaging procedures with both machines had been described previously^8–10^. In both machines, 6 × 6-mm macular cube scan was acquired from both eyes for all participants. The AngioVue uses a laser of 840 nm wavelength to capture 70,000 A-scans per second, and split-spectrum amplitude-decorrelation angiography algorithm was used to minimize motion artifact. The Cirrus HDOCT uses a light source of 840 nm wavelength to capture 68,000 A-scans per second. A proprietary real-time eye tracking system was used to minimize artifacts from blinking^8^.

### OCTA image analysis

**Figure 1:**
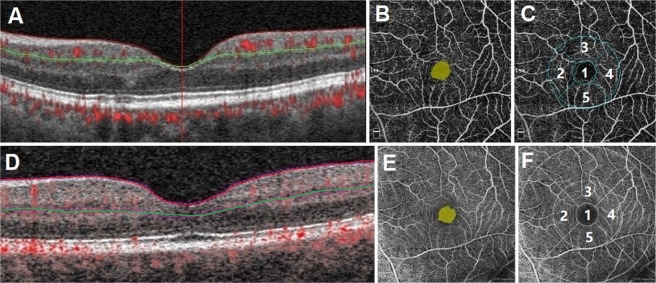
The optical coherence tomography angiography (OCTA) scans of a normal eye based on Angiovue and Cirrus HDOCT machines. OCTA scans within the superficial capillary plexus region (6×6-mm macular cube scan) using AngioVue (Fig A–C), and Cirrus HDOCT (Fig D–F). The demarcation of the superficial capillary plexus region is from the inner limiting membrane (marked by red line) to the inner plexiform layer (green line) (Fig A for Angiovue; Fig D for Cirrus HDOCT). Fig B and C show the foveal avascular zone (FAZ) area and vessel density (VD) measurement area in Angiovue, respectively. Fig E and F show the FAZ area and VD measurement area in Cirrus HDOCT, respectively. Foveal VD was measured from subfield 1 (1 mm diameter); while parafoveal VD was measured from the inner ring area (3 mm diameter) formed by subfields 2 to 5 (Fig C and F).

All scans were analyzed and measured automatically within the superficial capillary plexus (SCP) region, using the respective machines’ built-in review software. In the AngioVue (software version 2016.2.0.35), the SCP region was automatically segmented between an inner boundary 3 µm below the internal limiting membrane (ILM), and an outer boundary 15 µm below the inner plexiform layer (IPL) (Fig. 1A). In the Cirrus HDOCT (AngioPlex software, version10.0), the inner boundary of the SCP was set at the ILM, whereas the outer boundary was set at the level marked as 70% of the thickness between ILM and the outer plexiform layer (OPL) (Fig. 1D).

Quantitative analysis of the foveal avascular zone (FAZ) area within the SCP region was performed automatically using the AngioVue (Fig. 1B) and Cirrus software (Fig. 1E). The vessel density (VD) was defined as the total area of perfused vasculature per unit area in a region of measurement, which was also automatically calculated by AngioVue (Fig. 1C) and Cirrus HDOCT (Fig. 1F) review software. Foveal VD was measured from the central subfield zone 1 (1 mm diameter); while parafoveal VD was measured and averaged from the inner ring area (3 mm diameter) formed by subfields zone 2 to 5. The location and dimension of subfields 1 to 5 were the same between the built-in review software of AngioVue and Cirrus HDOCT.

### Clinical measurements and questionnaire

All participants underwent standardized systemic and ophthalmic examinations. Non-fasting venous blood samples were collected and analyzed for biochemical testing of serum glycated hemoglobin (HbA1c), glucose and total cholesterol. Diabetes was defined as either random glucose ≥11.1 mmol/L, HbA1c ≥ 6.5%, use of diabetic medication, or self-reported history. Hypertension was defined as either systolic blood pressure (BP) ≥ 140 mmHg, diastolic BP ≥ 90 mmHg, antihypertensive drugs usage, or self-reported history of hypertension. Hyperlipidemia was defined as either total cholesterol ≥6.2 mmol/L or use of lipid lowering medication. Spherical equivalent (SE) was calculated as the spherical value plus half of the negative cylinder value. Axial length (AL) was measured using non-contact partial coherence interferometry (IOL Master V3.01, Carl Zeiss Meditec AG, Jena, Germany).

A detailed interviewer-administered questionnaire was used to collect information including medication use, history of systemic and ocular disease, history of retinal surgery and laser treatment, as well as smoking status (classified as current and non-current smokers).

### Statistical analysis

Data from both eyes were included in the analysis if available. We first described the distribution of the FAZ area and VD parameters of each machine. The correlations between the parameters measured from both machines, were calculated using Pearson correlation coefficient. The agreement in FAZ area and VD measurements between both machines, was evaluated using intraclass correlation coefficients (ICC, based on absolute agreement model)^11^ and Bland-Altman plot^12^.

ICC values of 0.81 to 1.00 indicate almost perfect agreement, values of 0.61 to 0.80 indicate good agreement, values of 0.41 to 0.60 indicate moderate agreement, Values less than 0.40 indicate poor to fair agreement^13^. In Bland-Altman plot, the difference of two measurements (from both machines) was plotted against the average of two measurements. Proportional bias was investigated by testing whether the slope of the least squares regression line for difference of two measurements against the average of two measurements significantly differed from zero. Meanwhile, systemic bias was investigated by comparing the mean difference values with zero using one-sample *t* test^14^. The presence of proportional bias indicates that the differences between both machines are not constant throughout the range of measurements, whereas systemic bias indicates the differences are fixed throughout all measurements.

All statistical analyses were performed using Stata 13.0 (StataCorp LP, College Station, TX). The P value (2-sided) for significance was set at less than 0.05.

## Results

**Figure 2:**
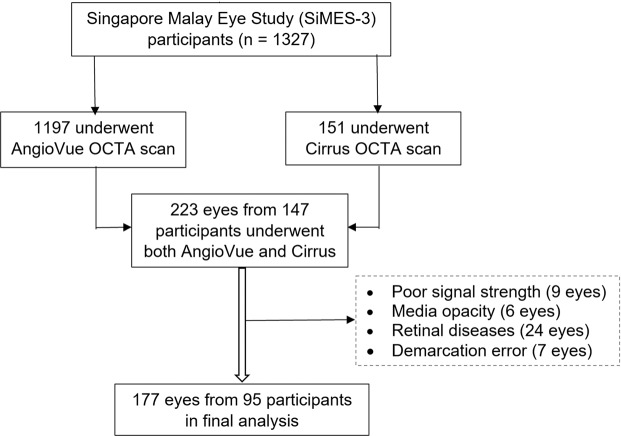
Flow chart illustrating inclusion of study participants.

Of the 223 eyes from the original 147 participants who underwent OCTA scans with the AngioVue and Cirrus HDOCT, 46 eyes were excluded (Fig. 2) due to poor signal strength, significant media opacity (6 eyes, e.g. dense cataract and large floater), retinal diseases (24 eyes, due to diabetic retinopathy, age-related macular degeneration, epi-retinal membrane, pigment epithelial detachment, wet age-related macular degeneration), and segmentation error made by the built-in review software (7 eyes). Thus, leaving 177 eyes from 95 participants in the final analysis. The mean age of included participants was 62.6 ± 6.8 years, and 51 (53.7%) of them were female. All participants were Malays.

Table 1 shows the systemic and ocular characteristics of study participants. 73 (76.8%) participants had hypertension, 48 (50.5%) had diabetes, and 68 (71.6%) participants had hyperlipidemia. 18 (19.0%) were current smokers. The mean spherical equivalent was 0.27 ± 1.74 diopter, and the mean axial length was 23.52 ± 1.00 mm.

**Table 1:** Characteristics of study participants.

Variables	n (%) or mean (SD)
Participants characteristics (n = 95)	
Age (years)	62.6 (6.8)
Female gender	51 (53.7)
Hypertension	73 (76.8)
Diabetes	48 (50.5)
Hyperlipidemia	68 (71.6)
Current smoker	18 (19.0)
Ocular characteristics (n = 177 eyes)	
Spherical equivalent, diopter	0.27 (1.74)
Axial length (mm)	23.52 (1.00)

Data presented as number (%), except for age, spherical equivalent and axial length which were expressed as means (standard deviation).

The distribution and correlations between OCTA parameters in the SCP are presented in Table 2. The mean FAZ area was 0.38 ± 0.11 mm^2^ in Angiovue, and 0.30 ± 0.10 mm^2^ in Cirrus HDOCT. The mean foveal VD was 0.29 ± 0.05 in Angiovue, and 0.20 ± 0.07 in Cirrus HDOCT. The mean parafoveal VD was 0.50 ± 0.04 and 0.43 ± 0.04 in the AngioVue and Cirrus HDOCT, respectively. Significant linear correlations albeit small effect estimates were observed between the parameters from both machines, this included the FAZ area (*r* = 0.4282, P < 0.001), foveal VD (*r* = 0.4985, P < 0.001) and parafoveal VD (*r* = 0.2022, P = 0.007).

**Table 2:** Distribution and correlations of OCTA parameters (within the superficial capillary plexus region) based on measurements from AngioVue and Cirrus HDOCT machines.

OCTA parameters	AngioVue		Cirrus		Pearson’s correlation coefficient, *r*	P value
	Mean	SD	Mean	SD		
FAZ area, mm^2^	0.38	0.11	0.30	0.10	0.4282	<0.001
Vessel density						
Fovea	0.29	0.05	0.20	0.07	0.4985	<0.001
Parafovea*	0.50	0.04	0.43	0.04	0.2022	0.007
Nasal subfield	0.50	0.05	0.43	0.04	0.1588	0.035
Superior subfield	0.51	0.05	0.44	0.04	0.2658	<0.001
Temporal subfield	0.51	0.04	0.42	0.05	0.0908	0.230
Inferior subfield	0.49	0.05	0.43	0.04	0.1393	0.065

OCTA: optical coherence tomography angiography, SD: standard deviation, FAZ: foveal avascular zone.

*Parafoveal parameter was averaged from measurements of nasal, superior, temporal and inferior macular subfields.

**Figure 3:**
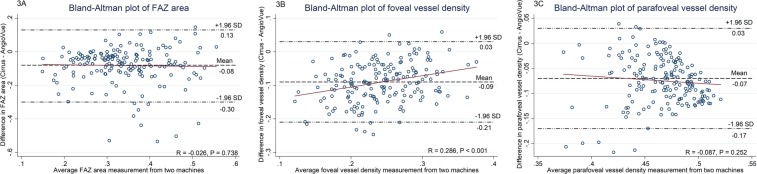
Bland-Altman plots showing agreement in (**A**) foveal avascular zone (FAZ) area, (**B**) foveal vascular density (VD), and (**C**) parafoveal VD measurements between Angiovue and Cirrus HDOCT machines. FAZ: foveal avascular zone.

Table 3 further shows the agreement in parameters between AngioVue and Cirrus HDOCT. The ICC values of FAZ area, foveal VD were 0.33 and 0.22, respectively; while parafoveal VD was 0.07, indicating poor to fair agreement between these measurements. Measurements from the Cirrus HDOCT were consistently lower than those by AngioVue, with mean differences of −0.08 mm^2^ (95% limits of agreement [LOA], −0.30 to 0.13) in FAZ area (Fig. 3A), −0.09 (95% LOA, −0.21 to 0.03) in the foveal VD (Fig. 3B), and −0.07 (95% LOA, −0.17 to 0.03) in the parafoveal VD (Fig. 3C). Systemic bias was found in the measurements of the FAZ area, foveal VD, parafoveal VD, as well as VD in all parafoveal subfields (all P < 0.001). On the other hand, the VD differences between both machines for measurements of foveal VD, nasal, superior and temporal macular subfields significantly correlated with the average of both machines’ measurements (all P ≤ 0.029) and thus indicated presence of proportional bias.

**Table 3:** Agreement analysis of OCTA parameters (within the superficial capillary plexus region) between AngioVue and Cirrus HDOCT machines.

OCTA parameters	ICC (95% CI)	Mean difference† (95% LOA)	P-value^	Systemic bias	P-value^#^	Proportional bias
FAZ area, mm^2^	0.33 (0.05, 0.53)	−0.08 (−0.30, 0.13)	<0.001	Yes	0.738	No
Vessel density						
Fovea	0.22 (−0.09, 0.50)	−0.09 (−0.21, 0.03)	<0.001	Yes	<0.001	Yes
Parafovea*	0.07 (−0.05, 0.21)	−0.07 (−0.17, 0.03)	<0.001	Yes	0.252	No
Nasal subfield	0.06 (−0.05, 0.19)	−0.07 (−0.18, 0.04)	<0.001	Yes	<0.001	Yes
Superior subfield	0.11 (−0.06, 0.29)	−0.07 (−0.17, 0.03)	<0.001	Yes	0.007	Yes
Temporal subfield	0.03 (−0.04, 0.11)	−0.09 (−0.22, 0.03)	<0.001	Yes	0.029	Yes
Inferior subfield	0.07 (−0.05, 0.20)	−0.06 (−0.19, 0.06)	<0.001	Yes	0.064	No

OCTA: optical coherence tomography angiography, ICC: intraclass correlation coefficient, CI: confidence interval, LOA: limits of agreement, FAZ: foveal avascular zone.

^P-value of one sample t-tests (comparing between mean difference and zero value) to indicate presence of systemic bias.

^#^P-value of regression line on difference against average of measurements from Cirrus HDOCT and AngioVue machines to indicate presence of proportional bias.

^†^Mean difference was determined from Cirrus HDOCT measurement minus AngioVue measurement.

*Parafovea parameter was averaged from measurements of nasal, superior, temporal and inferior subfields.

Further sensitivity analyses which randomly selected one study eye from each participant showed largely similar results as the original results which used both eyes (Supplementary Tables 1 & 2).

## Discussion

In this study, we evaluated the agreement in OCTA parameters (within the SCP region) between two commonly used OCTA modalities. Our results demonstrated that the measurements of the FAZ and VD parameters from the Cirrus HDOCT were consistently lower than AngioVue, and the agreements in these parameters between the two machines were generally poor to fair. These data suggest that alternating use between the two machines may not be recommended, especially in multi-center clinical trials and long-term follow up visits.

The FAZ is the central area of macula without blood vessels, but surrounded by a continuous network of capillaries^15^. It provides clinically useful information for macular ischemia^16^. It is also a significant predictor for visual acuity in DR, retinal vein occlusion^17^ and glaucoma^18^. The advent of OCTA provided new ways of quantifying the FAZ area^16^, In our study, the agreement in the FAZ area between the two OCTA machines was poor. Consistent with our finding, previous studies also reported significant differences in the FAZ area measurement from different machines^8,15,19^. Two factors might contribute to this poor agreement. Firstly, the segmentation of the FAZ boundary in each machine is different^16^. For instance, in AngioVue, automated segmentation of the SCP was set at an inner boundary at 3 µm below the ILM and outer boundary at 15 µm below the IPL. In Cirrus HDOCT, the SCP region’s inner boundary was set at the ILM, whereas the outer boundary was an estimated boundary of the IPL, which was marked at the ‘level’ where it was 70% of the thickness between ILM and the OPL. Secondly, the density of B-scans in the same scan was different between the two machines as well. In the 3 × 3-mm area, the AngioVue captures 304 B-scans, while the Cirrus HDOCT captures 245 B-scans. This might have also resulted in the different measurements observed between the two machines^15^. The overall poor agreement of FAZ area indicates that the results from the two machines cannot be interpreted interchangeably.

Quantification of the macular VD using OCTA is a potential imaging marker that may be deployed in clinical practice in the future. In our study, the agreement between the two machines for parafoveal VD measurement was poor to fair. This was consistent with most previous studies^8,15^. The poor agreement of the VD may be partially due to the different segmentation boundaries of retinal layer in different OCTA machines (as described above in Methods). The slightly different ‘slabbed’ layer might result in different VD measurement^15^. On the other hand, two previous studies reported good reproducibility of the VD measurement across different machines^19,20^. However, these studies had very small sample size (n ≤ 24 eyes), and might be subjected to bias.

The strengths of our study include the stringent quality control performed on all OCTA scans. All B-scans were checked for alignment and segmentation errors. Images with segmentation errors, poor signal strength or retinal diseases which might potentially affect the FAZ and VD measurements, were excluded. This robust data preparation process further substantiates the validity of our results. However, a few limitations should be noted. First, we only compared parameters within the SCP region between AngioVue and Cirrus HDOCT. This was because measurements in other retinal layers were not available from the Cirrus AngioPlex review software when this study was conducted. Second, even though we only evaluated parameters of the same measurement area from both machines, it cannot be entirely ruled out that the scans might not overlap perfectly due to the potential slight difference in fixation pattern of study eye from one machine to another, especially given that the ‘registered fixation pattern’ in Angiovue could not be transferred to Cirrus HDOCT, and vice versa. Nevertheless, as all scans were thoroughly checked for scan alignment (i.e. centred at fovea), we expect this limitation to have minimal impact on the overall findings. Lastly, this study was restricted to eyes without retinal diseases, therefore generalizability of our results to eyes with pathologies may be limited.

In conclusion, in eyes without retinal diseases, between AngioVue and Cirrus HDOCT, the agreements in the FAZ and VD parameters were poor to fair. Our findings suggest that alternating use between these two machines may not be recommended.

## Supplementary information

Supplementary Tables
