# Supplementary material for: Agreement in Measures of Macular Perfusion between Optical Coherence Tomography Angiography Machines
Source: Sci Rep. 2020 May 20;10:8345. doi: 10.1038/s41598-020-65243-2 (PMC7239842; doi:10.1038/s41598-020-65243-2)
Supplement: Supplementary file 1 — Supplementary Tables [file 41598_2020_65243_MOESM1_ESM.pdf]

Agreement in Measures of Macular Perfusion between Optical Coherence Tomography Angiography Machines

Wei Dai[1], Miao-Li Chee[1], Shivani Majithia[1], Cong Ling Teo[1], Sahil Thakur[1], Ning Cheung[1,2], Tyler Hyungtaek Rim[1,2], Gavin S. Tan[1,2], Charumathi Sabanayagam[1,2], Ching-Yu Cheng[1,2,3], Yih-Chung Tham[1,2].

1. Singapore Eye Research Institute, Singapore National Eye Centre, Singapore.
2. Ophthalmology & Visual Sciences Academic Clinical Program (Eye ACP), Duke-NUS Medical School, Singapore.
3. Department of Ophthalmology, Yong Loo Lin School of Medicine, National University of Singapore, Singapore.

Supplementary Table 1. Distribution and correlation of OCTA parameters (within the superficial capillary plexus region) based on measurements from the AngioVue and Cirrus HDOCT *(with random selection of one eye from each participant)*.

OCTA parameters	AngioVue		Cirrus		Pearson's correlation coefficient, *r*	P value
	Mean	SD	Mean	SD		
FAZ area, mm^2	0.38	0.12	0.29	0.10	0.440	<0.001
Vessel density						
Fovea	0.29	0.05	0.20	0.07	0.533	<0.001
Parafovea*	0.50	0.04	0.43	0.04	0.209	0.042
Nasal subfield	0.50	0.05	0.43	0.04	0.108	0.299
Superior subfield	0.51	0.05	0.44	0.04	0.286	0.005
Temporal subfield	0.51	0.04	0.42	0.06	0.133	0.200
Inferior subfield	0.49	0.05	0.43	0.04	0.112	0.282

OCTA: optical coherence tomography angiography, SD: standard deviation, FAZ: foveal avascular zone.

*Parafoveal parameter was averaged from measurements of nasal, superior, temporal and inferior macular subfields.

Supplementary Table 2. Agreement analysis of OCTA parameters (within the superficial capillary plexus region) between the AngioVue and Cirrus HDOCT machines *(with random selection of one eye from each participant)*.

OCTA parameters	ICC (95% CI)	Mean difference† (95% LOA)	P-value^	Systemic bias	P-value#	Proportional bias
FAZ area, mm^2	0.32 (0.01, 0.55)	-0.09 (-0.32, 0.13)	<0.001	Yes	0.517	No
Vessel density						
Fovea	0.25 (-0.10, 0.55)	-0.09 (-0.21, 0.03)	<0.001	Yes	0.018	Yes
Parafovea*	0.07 (-0.06, 0.22)	-0.08 (-0.17, 0.01)	<0.001	Yes	0.673	No
Nasal subfield	0.04 (-0.05, 0.16)	-0.07 (-0.19, 0.04)	<0.001	Yes	0.371	No
Superior subfield	0.12 (-0.07, 0.33)	-0.07 (-0.17, 0.03)	<0.001	Yes	0.017	Yes
Temporal subfield	0.04 (-0.05, 0.16)	-0.10 (-0.23, 0.03)	<0.001	Yes	<0.001	Yes
Inferior subfield	0.06 (-0.06, 0.19)	-0.06 (-0.19, 0.06)	<0.001	Yes	0.475	No

OCTA: optical coherence tomography angiography, ICC: intraclass correlation coefficient, CI: confidence interval, LOA: limits of agreement, FAZ: foveal avascular zone.

^P-value of one sample t-tests (comparing between mean difference and zero value) to indicate presence of systemic bias.

#P-value of regression line on difference against average of measurements from Cirrus HDOCT and AngioVue machines to indicate presence of proportional bias.

†Mean difference was determined from Cirrus HDOCT measurement minus AngioVue measurement.

*Parafoveal parameter was averaged from measurements of nasal, superior, temporal and inferior macular subfields.
